# Effects of rosuvastatin combined with fasudil therapy on rabbits with dyslipidemia

**DOI:** 10.1186/s12944-015-0050-1

**Published:** 2015-05-28

**Authors:** Zhiming Li, Hua Lian, Qin Liang, Fanfang Zeng, Dongdan Zheng

**Affiliations:** Department of Cardiology, Huizhou Municipal Central Hospital, 41st Eling North RD, Huicheng District, Huizhou, 516000 China; Department of Cardiology, Shenzhen Sun Yat-sen Cardiovascular Hospital, Shenzhen, 518000 China; Department of Cardiology, The Easten Hospital of the First Affiliated Hospital of Sun Yat-sen University, 58 Zhongshan Road 2, Guangzhou, 510080 China

**Keywords:** Dyslipidemia, Endothelial function, RhoA-associated kinase

## Abstract

**Background:**

Present study was conducted to investigate the effects of rosuvastatin combined with fasudil on rabbits with dyslipidemia.

**Methods:**

Dyslipidemia model of rabbits were produced by prescribing atherogenic diet for 2 weeks. Thereafter, 40 rabbits with dyslipidemia were randomly and evenly divided into four groups as follow: untreated group (orally prescribed 3 ml of normal saline), rosuvastatin group (orally prescribed 3 mg/kg body weight daily, dissolved in 3 ml of normal saline), fasudil group (intravenously prescribed 0.5 mg/kg body weight daily, dissolved in 3 ml of normal saline), and combined group (the same doses of rosuvastatin and fasudil as aforementioned). At baseline, 2 weeks of dyslipidemia establishment and 2 weeks of medical therapy, fasting venous blood was drawn for laboratory examination.

**Results:**

After 2 weeks’ atherogenic diet treatment, lipid disorders and impaired fasting glucose were observed. Systemic inflammation and oxidation were also promoted as revealed by increased serum levels of high sensitive C-reactive protein (Hs-CRP) and malondialdehyde (MDA). Notably, endothelial function has been impaired significantly as reflected by decreased nitric oxide (NO) production and increased serum asymmetric dimethylarginine (ADMA) level. RhoA associated kinase (ROCK) activity was also profoundly enhanced (*P* < 0.05). Inter-group comparisons showed that when compared to untreated group, modest improvements of endothelial function, inflammation and oxidation were observed in rosuvastatin and fasudil groups (*P* > 0.05). These benefits were improved more prominently in combined group (*P* < 0.05). Intra-group comparisons also showed that when compared to 2 weeks of dyslipidemia, slight improvement of endothelial function, inflammation and oxidation in rosuvastatin and fasudil groups were observed (*P* > 0.05). The improvements were more prominent in the combined groups (*P* < 0.05).

**Conclusion:**

Rosuvastatin combined with fasudil conferred synergistic effects on endothelium-protection and inflammation- and oxidation-amelioration in the setting of early stage of dyslipidemia.

## Introduction

Dyslipidemia, featured by increased serum level of low density lipoprotein cholesterol (LDL-C), is one of the most important risk factors for atherosclerosis and atherosclerotic cardiovascular disease (ASCVD) worldwide [1, 2]. In the early stage of dyslipidemia, endothelium becomes dysfunction in accompany with systemic inflammation and oxidation [3, 4]. Therefore, effectively treating dyslipidemia at an early stage by lifestyle modification or medicines should be beneficial for restoring endothelial function and preventing atherosclerosis progression [2].

With regard to lipid-lowering medicines, statins is the most commonly used currently. Other than lowering cholesterol, statins has other efficacies now universally known as pleiotropic effects which are largely dependent upon its potent effects on inhibiting isoprenylation of the small GTP-binding proteins during cholesterol biosynthesis [5, 6]. RhoA is the key member of small GTP-binding proteins and through activating its main downstream effector, named Rho associated kinase (ROCK), RhoA exerts multiple adverse effects on cardiovascular system [7, 8]. Fasudil, the specific inhibitor of ROCK, has been found potential to protect endothelium and inhibit inflammatory cells infiltration in the basic researches [9, 10]. However, no consistent clinical benefits have been achieved in previous studies with the use of fasudil on ASCVD therapy. Regarding the potent efficacies of statins and fasudil on vascular system, it is reasonable and rational to postulate that statins combined with fasudil may render synergistic effects on restoring endothelial function and preventing atherosclerosis progression. However, the evidence is lacking. Therefore, we conducted a basic research using rabbits with dyslipidemia model and giving different therapeutic strategies to address our hypothesis.

## Methods

### Animal preparations

The protocol for dyslipidemia model production was approved by the Ethic Committee of the First Affiliated Hospital of Sun Yat-sen University. Totally 50 male New Zealand White rabbits, weighing 1.3–1.5 kg and 5–8 weeks old, were used in present study. After 1 week’s accommodation, according to previous research [11], 40 rabbits, used as dyslipidemia model production, were randomly selected and treated with 50 g per kilogram body weight per day of standard chow diet enriched with 0.5 % cholesterol (Sigma Aldrich, St. Louis, MO, EUA) for 2 weeks. The other 10 rabbits, used as the control group, were given standard chow of 50 g per kilogram body weight per day. All animals received water ad libitum.

### Therapeutic strategies

After dyslipidemia model was successfully established as revealed by LDL-C elevation, 40 rabbits with dyslipidemia were randomly and evenly divided into four groups: untreated group (orally prescribed 3 ml of normal saline), rosuvastatin group (orally prescribed 3 mg/kg body weight daily, dissolved in 3 ml of normal saline), fasudil group (intravenously prescribed 0.5 mg/kg body weight daily, dissolved in 3 ml of normal saline), and combined group (the same doses of rosuvastatin and fasudil as aforementioned). The therapeutic duration was 2 weeks.

### Laboratory examination

At baseline, 2 weeks of dyslipidemia production and 2 weeks of medical therapy, fasting venous blood was drawn for laboratory examination. Lipid profiles including triglyceride (TG), total cholesterol (TC), high density lipoprotein cholesterol (HDL-C) and LDL-C, fasting blood glucose (FBG), liver enzymes including alanine aminotransferase (ALT) and aspartase aminotransferase (AST) were detected by Automatic Biochemistry Analyzer (Beckman coulter UniCel DxC 800 Synchron). Parameters of endothelial function including nitric oxide (NO) production (ELISA kit, Nanjing Jiancheng Bioengineering Institute) and serum level of asymmetric dimethylarginine (ADMA, ELISA kit, Shanghai Ying-gong Industrial Company) were assessed in accordance to the manufacture’s introduction. Serum levels of high sensitive C-reactive protein (Hs-CRP Assay Kit, Immune-turbidimetry method, Nanjing Jiancheng Bioengineering Institute) and malondialdehyde (MDA Assay Kit, TBA method, Nanjing Jiancheng Bioengineering Institute) were measured to evaluate the changes of systemic inflammation and oxidation over time. Serum ROCK activity was detected by enzyme-linking immune-absorbent assay (ELISA kit, Yuping BioMedical Company, Shanghai, China).

### Statistical analyses

Data were expressed as means ± S.E.M., and inter-group and intra-group comparisons were analyzed by one-way ANOVA followed by Dunnett’s multiple comparison test or by student *t*-test when appropriately using SPSS 19.0 statistical analysis program. *P* < 0.05 was considered as significant.

## Results

### Changes of parameters before and after dyslipidemia establishment

**Fig. 1:**
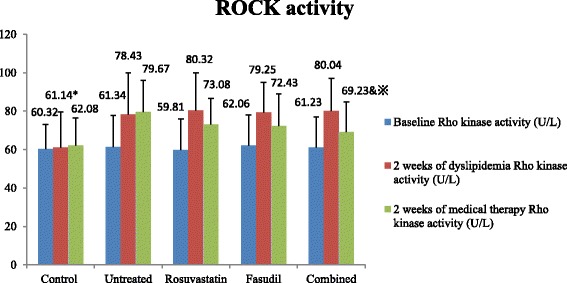
Inter- and intra-groups comparisons of ROCK activity. Denote: * *P* < 0.05 versus other groups at the same time point; & *P* < 0.05 versus the Untreated groups at the same time point; ^※^ *P* < 0.05 versus 2 weeks of dyslipidemia in the same group

**Fig. 2:**
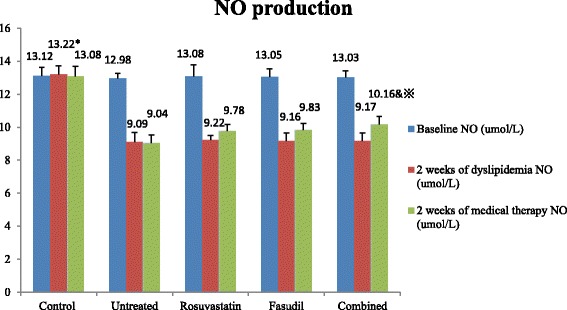
Inter- and intra-groups comparisons of NO production. Denote: * *P* < 0.05 versus other groups at the same time point; & *P* < 0.05 versus the Untreated groups at the same time point; ^※^ *P* < 0.05 versus 2 weeks of dyslipidemia in the same group

After 2 weeks of atherogenic diet treatment, inter-group differences were compared. As presented in Table 1, after 2 weeks of atherogenic diet treatment, lipid disorders and impaired fasting glucose were observed as indicated by increased serum levels of TG, TC, LDL-C and FBG. Systemic inflammation and oxidation were also promoted by atherogenic diet treatment as revealed by increased serum levels of Hs-CRP and MDA. Notably, with 2 weeks of dyslipidemia, endothelial function has significantly impaired as reflected by profoundly decreased NO production (Fig. 2) and significantly increased serum level of ADMA. ROCK activity was also profoundly enhanced as shown in Fig. 1. Compared to the control group, all the between-group differences were statistically significant (*P* < 0.05).

**Table 1:** Inter- and intra-groups comparison of parameters

Variables	Control	Untreated	Rosuvastatin	Fasudil	Combined
At baseline					
TG (mmol/L)	1.05 ± 0.11	1.07 ± 0.10	1.06 ± 0.11	1.06 ± 0.12	1.04 ± 0.10
TC (mmol/L)	3.37 ± 0.25	3.37 ± 0.20	3.34 ± 0.22	3.37 ± 0.21	3.39 ± 0.23
LDL-C (mmol/L)	1.99 ± 0.14	1.98 ± 0.12	1.99 ± 0.20	2.00 ± 0.19	1.97 ± 0.12
HDL-C (mmol/L)	1.05 ± 0.04	1.07 ± 0.03	1.05 ± 0.05	1.06 ± 0.04	1.06 ± 0.03
FBG (mmol/L)	5.83 ± 0.16	5.85 ± 0.15	5.80 ± 0.14	5.80 ± 0.16	5.82 ± 0.12
ALT (U/L)	33.7 ± 5.4	37.6 ± 3.8	34.1 ± 4.2	37.1 ± 4.9	35.6 ± 3.7
AST (U/L)	39.1 ± 4.0	37.3 ± 3.2	35.3 ± 3.1	37.5 ± 4.0	36.5 ± 3.0
Hs-CRP (mg/L)	2.62 ± 0.24	2.59 ± 0.15	2.66 ± 0.22	2.61 ± 0.17	2.63 ± 0.16
MDA (nmol/L)	0.99 ± 0.16	0.97 ± 0.15	0.93 ± 0.15	0.93 ± 0.15	0.97 ± 0.14
ADMA (nmol/L)	73.25 ± 10.09	72.32 ± 10.32	70.16 ± 11.08	71.43 ± 11.23	71.89 ± 11.45
2 weeks of dyslipidemia					
TG (mmol/L)	1.06 ± 0.13^*^	2.14 ± 0.20	2.18 ± 0.21	2.18 ± 0.16	2.16 ± 0.20
TC (mmol/L)	3.41 ± 0.32^*^	5.89 ± 0.42	5.87 ± 0.33	5.83 ± 0.32	5.88 ± 0.35
LDL-C (mmol/L)	1.98 ± 0.14^*^	3.83 ± 0.42	3.82 ± 0.40	3.84 ± 0.32	3.80 ± 0.26
HDL-C (mmol/L)	1.05 ± 0.03	1.01 ± 0.02	1.00 ± 0.04	1.01 ± 0.03	1.02 ± 0.02
FBG (mmol/L)	5.98 ± 0.15^*^	6.24 ± 0.16	6.29 ± 0.14	6.30 ± 0.15	6.27 ± 0.13
ALT (U/L)	36.4 ± 5.5	37.1 ± 4.2	36.4 ± 5.0	39.2 ± 4.7	37.7 ± 4.8
AST (U/L)	38.2 ± 4.7	38.6 ± 3.3	37.9 ± 3.2	37.5 ± 3.3	39.1 ± 3.6
Hs-CRP (mg/L)	2.73 ± 0.14^*^	7.28 ± 1.15	7.39 ± 1.06	7.52 ± 1.05	7.40 ± 1.08
MDA (nmol/L)	0.99 ± 0.12^*^	4.08 ± 0.40	4.15 ± 0.36	3.99 ± 0.44	4.04 ± 0.37
ADMA (nmol/L)	74.25 ± 10.33^*^	87.34 ± 10.08	87.17 ± 11.24	88.62 ± 10.25	86.60 ± 10.45
2 weeks’ medical therapy					
TG (mmol/L)	1.05 ± 0.11^*^	2.18 ± 0.16	2.09 ± 0.11	2.16 ± 0.13	2.09 ± 0.12
TC (mmol/L)	3.44 ± 0.30^*^	5.92 ± 0.36	5.16 ± 0.26^**^	5.90 ± 0.34	5.18 ± 0.33^**^
LDL-C (mmol/L)	1.99 ± 0.17^*^	3.86 ± 0.35	3.23 ± 0.21^**^	3.85 ± 0.30	3.20 ± 0.22^**^
HDL-C (mmol/L)	1.06 ± 0.04	1.00 ± 0.03	1.03 ± 0.03	1.03 ± 0.04	1.05 ± 0.02
FBG (mmol/L)	5.92 ± 0.16^*^	6.21 ± 0.18	6.10 ± 0.17	6.13 ± 0.14	6.10 ± 0.15
ALT (U/L)	38.2 ± 2.7	37.8 ± 3.2	36.7 ± 2.5	36.9 ± 2.6	35.7 ± 3.3
AST (U/L)	38.4 ± 3.5	38.9 ± 3.0	37.5 ± 3.6	37.0 ± 3.9	37.9 ± 3.6
Hs-CRP (mg/L)	2.76 ± 0.11^*^	7.33 ± 1.12	6.92 ± 1.00	7.00 ± 1.02	6.34 ± 0.96^***,****^
MDA (nmol/L)	0.97 ± 0.15^*^	4.11 ± 0.36	3.95 ± 0.30	3.91 ± 0.24	3.36 ± 0.35^***,****^
ADMA (nmol/L)	70.46 ± 9.38^*^	87.88 ± 10.23	83.35 ± 9.68	83.07 ± 10.08	80.14 ± 10.20^***,****^

^*^ *P* < 0.05 versus other groups, ^**^ *P* < 0.05 versus Untreated and Fasudil groups, ^***^ *P* < 0.05 versus Untreated groups, ^****^ *P* < 0.05 versus 2 weeks of dyslipidemia in the same group

### Inter-group comparison of parameters after medical therapy

After 2 weeks of medical therapy, inter-group differences regarding the effects of different therapeutic strategies were evaluated and compared. As shown in Table 1, Figs. 1 and 2, lipid profiles were improved in the rosuvastatin and combined groups but not in the untreated and fasudil groups (*P* < 0.05). Serum level of FBG was similarly declined in the 3 medical therapy groups, however, no significant between-group difference was observed when compared to the untreated group. Modest between-group differences were observed in the parameters of Hs-CRP, MDA, NO production, ADMA and ROCK activity in the rosuvastatin and fasudil groups when compared to the untreated group (*P* > 0.05). Notably, rosuvastatin combined with fasudil therapy had synergistic effects as reflected by the more prominent improvement in the parameters of Hs-CRP, MDA, NO production, ADMA and ROCK activity when compared to the untreated group (*P* < 0.05).

### Intra-group comparison of parameters after medical therapy

Intra-group comparisons of parameters were also compared. As shown in Table 1, Figs. 1 and 2, the parameters of Hs-CRP, MDA, NO production, ADMA and ROCK activity were slightly improved in the rosuvastatin and fasudil groups when compared to those at 2 weeks of dyslipidemia (*P* > 0.05). However, the improvement in the parameters of Hs-CRP, MDA, NO production, ADMA and ROCK activity were more prominent in the combined groups when compared to those of 2 weeks of dyslipidemia (*P* < 0.05), suggesting that rosuvastatin combined with fasudil therapy had synergistic effects on improving endothelial dysfunction, inflammation and oxidation induced by dyslipidemia.

## Discussion

Dyslipidemia is prevalent worldwide and is one of the most important risk factors for multiple diseases such as ASCVD. Endothelial dysfunction is implicated in the early stage of dyslipidemia as well as in the process of atherosclerosis development [12, 13]. Therefore, treating dyslipidemia effectively is of clinical importance. Results from present research show that rosuvastatin not only could ameliorate dyslipidemia but also could improve endothelial function as well as systemic inflammation and oxidation, which strongly supporting the cardio-protective effects of rosuvastatin therapy. Moreover, although no effects on lipid-modification, fasudil therapy is effective on improving endothelial function, inflammation and oxidation. Expectedly and importantly, fasudil combined with rosuvastatin therapy confers synergistic effects on vascular system in the early stage of dyslipidemia which should have important clinical relevance.

Knowingly [14], LDL-C elevation is detrimental to endothelium and could promote systemic inflammation and oxidation through multiple mechanisms. Therefore, decreasing LDL-C level by statins therapy is of clinical importance in preventing cardiovascular events. Other than lipid-lowering effects, statins has other efficacies which are predominantly associated with its effects on attenuating small GTP-binding proteins isoprenylation during cholesterol biosynthesis. As is well known that RhoA isoprenylation, the most commonly studied small GTP-binding protein, could lead to its downstream target ROCK activation thereby eliciting multiple adverse effects such as vessel constriction, inflammatory cells migration and infiltration, platelet activation, and endothelial dysfunction [15, 16]. Therefore, not only decreasing RhoA isoprenylation is critical, inhibiting ROCK activity is also crucial in preventing atherosclerosis development and reducing cardiovascular events. For example, Naoki Sawada et al. reported that Y-27632, a specific ROCK inhibitor, might be an effective therapeutic strategy for treating vascular proliferative disorders and hypertension [17]. In addition, results from Anju Nohria showed that inhibition of the Rho/ROCK signaling pathway by fasudil should provide a useful strategy to restore NO bioavailability in humans with atherosclerosis [18]. Taken together, we postulated that statins combined with ROCK antagonist could provide synergistic effects in treating dyslipidemia and its associated inflammation and oxidation, and results from our preliminary research supported this hypothesis. Of note, in our present research, although fasudil had no benefit on dyslipidemia modification, nonetheless, we observed that endothelial function as well as systemic inflammation and oxidation were all improved after 2 weeks of fasudil therapy, which strongly suggesting that the benefits derived from fasudil therapy was independent of lipid-lowering. Notably, these benefits were further enhanced by combined therapy which we considered was associated with further attenuation of ROCK activity. In light of previous reports [6, 19–21], we considered that the two following mechanisms might at least partially explain our findings. In the first place, by reducing RhoA isoprenylation during cholesterol biosynthesis, rosuvastatin could robustly inhibit ROCK activation thereby enhancing endothelial function and ameliorating systemic inflammation and oxidation. On the other hand, since fasudil is a specific and potent antagonist for ROCK, therefore, as adjunctive to inhibiting ROCK activation by rosuvastatin therapy, fasudil therapy could block the downstream effects provoking by already-activated RhoA/ROCK signaling pathway. Taken together, through different and complemented mechanisms, rosuvastatin combined with fasudil therapy conferred synergistic and protective effects on vascular system in rabbits with dyslipidemia. O-linked N-acetylglucosamine (O-GlcNAc) is a reversible post-translational modification of serines/threonines substrate and has been found associated with the development of dyslipidemia and other cardiovascular diseases [22, 23]. Therefore, detecting the change of O-GlcNAc should provide mechanisms regarding the additive benefits of combined therapy on rabbits with dyslipidemia. Thus, it is the potential limitation of our present research for not investigating the change of O-GlcNAc before and after therapy and in the future it is important and warranted to further investigate the relationship between O-GlcNAc and the application of rosuvastatin and fasudil therapy.

Interestingly, we observed that dyslipidemia resulted in fasting blood glucose elevation, and with rosuvastatin, fasudil or combined therapy, fasting blood glucose was modestly reduced. Whether this finding had clinical implications regarding the development of metabolism syndrome or diabetes mellitus in the setting of dyslipidemia needed further investigation. Moreover, concerning the safety of rosuvastatin and fasudil therapy, liver enzyme was serially detected and no significant elevations of ALT and AST were observed, which indicated that 2 weeks of rosuvastatin and fasudil therapy was no harm to liver function.

## Conclusion

Preliminary data from our present research revealed that rosuvastatin combined with fasudil therapy conferred synergistic effects on endothelium-protection and inflammation- and oxidation-amelioration in the setting of early stage of dyslipidemia. Further study is warranted to investigate whether these efficacies could translate into clinical benefits.
